# Treatment of Leg Veins for Restless Leg Syndrome: A Retrospective Review

**DOI:** 10.7759/cureus.4368

**Published:** 2019-04-02

**Authors:** Swaminathan Sundaresan, Michael R Migden, Sirunya Silapunt

**Affiliations:** 1 Dermatology, University of Texas Medical Branch, Galveston, USA; 2 Dermatology, Head and Neck Surgery, The University of Texas MD Anderson Cancer Center, Houston, USA; 3 Dermatology, University of Texas McGovern Medical School, Houston, USA

**Keywords:** restless leg syndrome, superficial venous reflux, endovenous radiofrequency ablation, ultrasound-guided foam sclerotherapy, chronic venous insufficiency

## Abstract

Background: Restless leg syndrome (RLS) and chronic venous insufficiency (CVI) share similar circadian timings and epidemiological characteristics.

Objective: The objective of the study was to investigate whether treating superficial venous reflux (SVR) improves the RLS severity in patients with CVI and whether there is an association of the RLS severity with the number of refluxed veins.

Materials and methods: Patients with RLS and duplex ultrasound-proven SVR were identified from a database of 134 patients. All patients underwent endovenous radiofrequency ablation and ultrasound-guided foam sclerotherapy. International RLS (IRLS) rating scale questionnaires were reviewed to assess pre- and post-intervention RLS status.

Results: Thirty-five patients were identified. The average baseline IRLS score was 19.83 (moderate RLS) and improved to 7.89 (mild RLS) after treatment (p < .0001), corresponding to 63% decrease in symptoms. Ten patients (29%) had a follow-up score of 0, indicating complete relief of RLS symptoms. Twenty patients (57%) had decreased IRLS scores of 10 points or more (i.e. 1 grade level of severity). Six patients had no improvement. There was no association of the RLS severity with the number of refluxed veins.

Conclusion: The study found that correcting SVR improves RLS symptoms, suggesting an association between CVI and RLS. Venous ultrasound study and intervention should be considered for potential patients.

## Introduction

Restless leg syndrome (RLS), also known as Willis-Ekbom disease, is characterized by an irresistible urge to move the legs to obtain relief from an uncomfortable sensation in the legs. RLS was first described in 1945 with an estimated prevalence of 5% [1]. Since its discovery, the prevalence has been found to be 3%-15% [2-4]. Despite its growing numbers and decades of investigative research RLS is poorly understood, which may be attributed to its broad range of presentations (see below) [5, 6]. Moreover, patients have difficulty even communicating and describing their symptoms [7].

RLS is reported more frequently in women with a near 2:1 female to male ratio. RLS can occur during pregnancy and be affected by trimester and number of parity. It has also been shown to have a positive correlation with increasing age [4]. Genetics plays a role as well, with up to 25% of first degree relatives of those with RLS reporting RLS-like symptoms [8]. RLS associations including female sex, multiparity, old age, and family history are also risk factors for chronic venous disease (CVD) [9].

The association between venous reflux and RLS was suggested by Kanter et al., who found those treated with sclerotherapy had improvement of RLS symptoms [10]. Later studies purported this association with findings of RLS symptoms in patients with primary and recurrent varicose veins [11, 12]. Treating superficial venous insufficiency has been shown to alleviate RLS symptoms [13]. We retrospectively studied the effect of endovenous radiofrequency ablation (RFA) and ultrasound-guided foam sclerotherapy (UGFS) in patients with superficial venous reflux to determine the effect on RLS symptoms and whether there is an association of the severity of RLS with the number of refluxed saphenous veins.

Diagnostic criteria for RLS

1. an urge to move the legs usually but not necessarily associated with feelings of discomfort

2. an urge to move the legs and unpleasant sensations are worse at rest

3. the symptoms are partially or totally relieved with movement

4. the symptoms worsen later in the day or at night

## Materials and methods

A database of 134 patients with duplex ultrasound-proven superficial venous reflux (SVR) from the Department of Dermatology at McGovern Medical School in Houston was screened for RLS. All patients underwent RFA of refluxed saphenous veins and UGFS of associated tributaries. Thirty-five patients were identified with RLS and included in the study. Data were collected on demographics (i.e., gender, age), past medical history and medications associated with RLS. Their international RLS rating scale questionnaires (IRLS) were reviewed and analyzed to assess the pre-intervention and post-intervention RLS status; mild (score 1-10); moderate (score 11-20); severe (score 21-30); very severe (score 31-40) (Table 1) [14].

**Table 1:** Restless Leg Syndrome (RLS) Rating Scale All items are scored and the sum of the item scores serves as the scale score. Scoring criteria are: mild (score 1-10); moderate (score 11-20); severe (score 21-30); very severe (score 31-40) [14].

	Questions	Answers (scores 0-4)
1	Overall, how would you rate the RLS discomfort in your legs or arms?	(4) Very severe (3) Severe (2) Moderate (1) Mild (0) None
2	Overall, how would you rate the need to move around because of your RLS symptoms?	(4) Very severe (3) Severe (2) Moderate (1) Mild (0) None
3	Overall, how much relief of your RLS arm or leg discomfort do you get from moving around?	(4) No relief (3) Slight relief (2) Moderate relief (1) Either complete or almost complete relief (0) No RLS symptoms; question does not apply
4	Overall, how severe is your sleep disturbance from your RLS symptoms?	(4) Very severe (3) Severe (2) Moderate (1) Mild (0) None
5	How severe is your tiredness or sleepiness from your RLS symptoms?	(4) Very severe (3) Severe (2) Moderate (1) Mild (0) None
6	Overall, how severe is your RLS as a whole?	(4) Very severe (3) Severe (2) Moderate (1) Mild (0) None
7	How often do you get RLS symptoms?	(4) Very severe (This means 6 to 7 days a week) (3) Severe (This means 4 to 5 days a week) (2) Moderate (This means 2 to 3 days a week) (1) Mild (This means 1 day a week or less) (0) None
8	When you have RLS symptoms how severe are they on an average day?	(4) Very severe (8 hours or more per 24 hour day) (3) Severe (3-8 hours per 24 hour day) (2) Moderate (1-3 hours per 24 hour day) (1) Mild (less than 1 hour per 24 hour day) (0) None
9	Overall, how severe is the impact of your RLS symptoms on your ability to carry out your daily affairs, for example carrying out a satisfactory family, home, social, school or work life?	(4) Very severe (3) Severe (2) Moderate (1) Mild (0) None
10	How severe is your mood disturbance from your RLS symptoms – for example angry, depressed, sad, anxious or irritable?	(4) Very severe (3) Severe (2) Moderate (1) Mild (0) None
All items are scored and the sum of the item scores serves as the scale score. Scoring criteria are: mild (score 1-10); moderate (score 11-20); severe (score 21-30); very severe (score 31-40) [14]		

Statistical analysis

In this retrospective review, the baseline and post-intervention IRLS scores were analyzed with univariate analysis (paired t-test) and significance was defined as p-value <.05. All data was analyzed with Microsoft Excel. A Student's t-test was used to assess IRLS scores between groups of patients with differing number of incompetent veins (i.e. 4 vs 3 vs 2).

## Results

**Figure 1:**
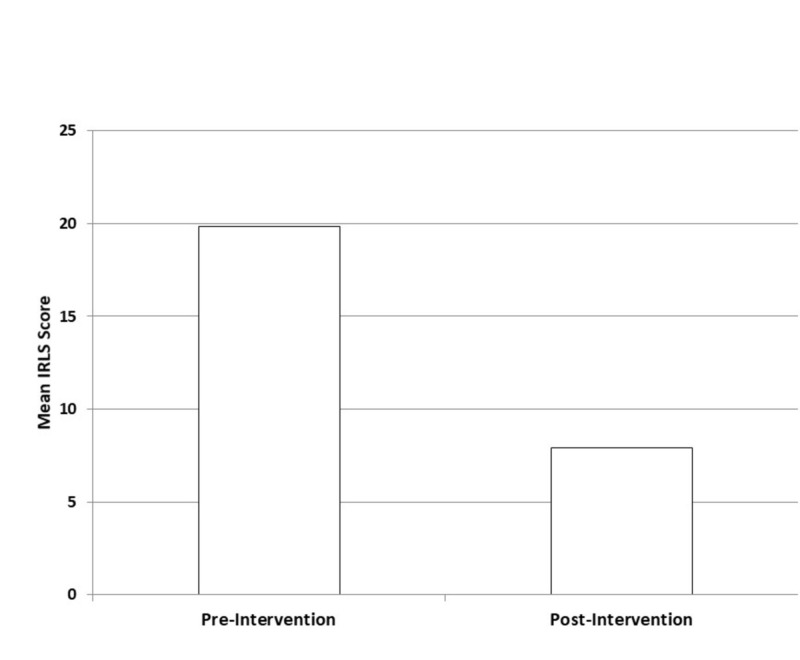
IRLS Rating Scores Before and After Intervention RLS - Restless leg syndrome, IRLS rating score - International RLS rating score

Thirty-five (26%) of the 134 patients screened in this study were positive for RLS. The 35 patients ranged in age from 38-81 years, with the typical RLS patient in the study being a female above the age of 60 years. The average IRLS score before intervention was 19.83 (moderate RLS, ±7.19) and improved to 7.89 (mild RLS, ±7.60) after treatment (p < .0001), corresponding to 63% decrease in symptoms (Figure 1). Ten patients (29%) with an average pre-treatment IRLS score of 21, corresponding to a severe grade of RLS symptoms, had a follow-up score of 0, indicating complete relief of RLS symptoms after treatment. Twenty patients (57%) had a decrease in IRLS scores of 10 points or more (i.e. 1 grade level of severity) and 21 (60%) patients improved to ‘mild’ disease. Males had a 63% decrease in their IRLS scores (19.63 to 7.25) compared to 59% for females post-intervention (19.89 to 8.07). Six of 35 (17%) patients did not report any improvement in their RLS. Of 35 patients, one had iron deficiency anemia treated with iron, one had kidney disorder, and two were taking antidepressants. Three of these four patients reported improvement of their RLS after intervention. Their average pre-treatment IRLS score was 21 (severe RLS, ±10) and post-treatment IRLS score was 11 (moderate RLS, ± 1) (p = 0.261). Three patients on dopamine agonists had an average pre-treatment IRLS score of 26 (severe RLS, ±3.61) and an average post-treatment IRLS score of 11 (moderate RLS, ±11) (p= .0751), with one patient reporting complete resolution of symptoms. Frictional blisters from compression bandages occurred in one patient. Postoperative discomfort requiring NSAIDs was reported in two patients. No major side effects or complications occurred during treatment or within the postoperative period. Patients with partial or no improvement were advised to follow-up with their primary care physician for evaluation of other causes and for potential medical treatment. A total of 16 (46%) patients were detected with four incompetent veins, nine (26%) patients had three incompetent veins, and 10 (29%) had two incompetent veins. There does not appear to be an association between baseline IRLS score and the number of veins that have reflux as the average pre-treatment IRLS score for patients with four vein reflux was 21.13 (severe RLS ±7.26) and for two vein reflux 17.7 (moderate ±6.0) (p = 0.23). Similar results were found when comparing three vein reflux 19.89 (moderate ±8.45) with two vein reflux 17.7 (moderate ±6.0) (p = 0.50) [15].

## Discussion

Restless leg syndrome is a condition characterized by an urge to move the legs, which typically becomes worse at night and at rest with partial relief by movement or walking [6]. Diagnosis of RLS is primarily based on history and physical examination, but it is imperative to rule out secondary causes. Secondary RLS has been attributed to distinct conditions such as iron deficiency anemia, pregnancy, end-stage renal disease, and venous disease [16, 17]. However, the direct etiology of RLS remains unclear.

Our study suggests that treating underlying venous disease can relieve the RLS symptoms. Considering that we observed RLS in 31.3% of those with duplex ultrasound-proven superficial venous reflux and a subsequent decrease of 63% in IRLS points after RFA and UGFS compared to baseline, we suggest that venous reflux is associated with RLS. However, we found no association of the severity of RLS with the number of refluxed saphenous veins (p > 0.2), as baseline IRLS score and the number of veins that have reflux as the average pre-treatment IRLS score were not statistically significant. Nevertheless, the question remains whether RLS is a symptomatic presentation that simultaneously occurs with chronic venous insufficiency (CVI) or whether CVI is a direct cause of RLS. If it is the latter, then what is the pathophysiological mechanism behind venous reflux and RLS?

One possible explanation centers around the accumulation of edema in patients with CVI. Impaired venous valves limit forward flow and result in reflux of blood that concentrates in the venous system [18]. The hydrostatic pressure is transmitted to venules and capillaries and results in an extravasation of fluid and decreases the interstitial fluid from receding back to the vasculature. The urge to move the legs may be rooted in the subconscious to contract skeletal muscles and propel venous blood to return back to the heart. When legs are placed in the recumbent position the decreasing venous pressure and the ebbing of fluid back into the vasculature could be the cause of the unpleasant sensations that plague RLS patients at night [13].

Personalized therapy can only be offered to individual cases of RLS when the exact etiology of RLS has been understood. If secondary causes are excluded, a large group of patients with idiopathic or primary RLS will remain. Primary (idiopathic) RLS is more common and typically has a family history with an autosomal dominant inheritance pattern proposed [8, 19, 20]. It is suspected to be a sensorimotor abnormality with deficiencies in the central dopaminergic pathways [8, 19, 21], since dopamine agonists have been found to improve symptoms [21]. One controlled trial showed improvement of RLS in 53.4% of patients treated with ropinirole, a dopamine agonist [22]. However, dopamine agonists were reported to cause the worsening of RLS symptoms in 48% of subjects who had at least six months of dopamine agonist treatment [23, 24]. The use of opioids, benzodiazepines and anticonvulsants have been reported for the treatment of RLS [25].

Our cohort included three patients who had been treated with dopamine agonist (i.e. ropinirole and pramipexole) but still had severe grade of RLS with an average IRLS score of 26. After RFA and UGFS treatment, all three patients had a decrease in IRLS scores with an average drop of 15 points (i.e. 1.5 grade levels of severity) and one patient reported complete resolution of symptoms. These changes were not significant (p=.0751), likely due to a small sample size (i.e. n=3).

Medications such as antidepressants and dopamine blocking agents have been reported to induce or exacerbate RLS [16, 25]. In our study, three of four patients who were on antidepressants and/or had disease comorbidities reported improvement in their RLS symptoms after the operative intervention. These three patients were found to have a 10 point drop in their IRLS scores from a pre-treatment score of 21 to a post-treatment score of 11 (p-value = 0.261). This suggests that RLS patients who do not improve with medication therapy may benefit from duplex ultrasound assessment for concurrent CVI.

## Conclusions

The decrease in IRLS points and significant improvement in the degree of severity of RLS after RFA and UGFS suggests an association between CVI and RLS. Venous procedures that correct superficial venous reflux should be considered as therapeutic treatment options with more definitive potential for patients with RLS.
